# Reliability and validity of a single item measure of quality of life scale for adult patients with cystic fibrosis

**DOI:** 10.1186/1477-7525-9-105

**Published:** 2011-11-25

**Authors:** Abebaw M Yohannes, Mary Dodd, Julie Morris, Kevin Webb

**Affiliations:** 1 Department of Health Professions, Research Institute for Health and Social Change, Manchester Metropolitan University, Elizabeth Gaskell Campus, Hathersage Road, M13 0JA, UK; 2 Department of Adult Cystic Fibrosis Unit, University of South Manchester, Wythenshawe Hospital, Manchester, Southmoor Road, M23 9LT, UK; 3 Department of Medical Statistics, 1st Floor, Education & Research Centre, University Hospital of South Manchester, Wythenshawe Hospital, Southmoor Road, Manchester, M23 9LT, UK

**Keywords:** Quality of life, single-item global scale, cystic fibrosis, reliability, concurrent validity, adult, HRQOL, CF-QOL

## Abstract

**Background:**

It is important to monitor health related quality of life in order to determine the efficacy of interventions and physical functioning of patients with cystic fibrosis in their daily activities. There is no a single-item global quality of life scale for routine clinical practice for adult patients with cystic fibrosis. We assessed the reliability and validity of a single-item global quality of life scale and compared with the Cystic Fibrosis Quality of Life Questionnaire (CF-QOL) for adult patients with cystic fibrosis.

**Method:**

121 **(**men = 66, women = 55) adult cystic fibrosis patients self-completed the CF-QOL, the Hospital Anxiety Depression Scale, and the single item global quality of life scale at the out patient clinic. 33 (17 women) completed the repeat questionnaires at home within two weeks. Socio-demographic characteristic and lung function data were extracted from the recent medical notes.

**Results:**

Mean (SD) age was 29.6 (8.9) years and mean (SD) forced expiratory volume in 1 second was 2.20 (0.94) litres. The test-retest reproducibility using the intra-class correlation coefficient (ICC) for the CF-QOL was 0.83, 95% confidence interval 0.68 to 0.91. The single item global quality of life ICC score was 0.78, 95% confidence interval 0.59 to 0.88. Concurrent validity of the single-item global quality of life was examined in relation to all items of the CF-QOL, frequent episodes of readmission, anxiety and depression (all, p < 0.01) were moderately correlated.

**Conclusion:**

The study provides preliminary evidence that the single-item quality of life scale is acceptable, valid and repeatable for adult patients with cystic fibrosis. It is a promising tool that can be easily incorporated into a routine clinical practice to assess patients' quality of life.

## Introduction

There are disease-specific validated health related quality of life (HRQOL) scales [1,2] that measure dimensions of health, not otherwise assessed by conventional lung function tests in order to determine the severity of the disease for adult patients with cystic fibrosis (CF). HRQOL scales are regarded as relevant endpoints to measure the efficacy of clinical drug trials and the benefits of rehabilitation for adult CF patients [3,4]. They also provide additional information that is specific to an individual and not captured, for example, by lung function tests or other endpoints.

HRQOL scales provide the overall impact of the disease and to gain further insight from the patients' perspective. The most commonly used disease-specific HRQOL scales for patients with cystic fibrosis are the Cystic Fibrosis Quality of Life Scale (CF-QOL) with 52 items with eight quality of life domains [1] and the Cystic Fibrosis Questionnaire Revised (CFQ-R) with 50 items with nine quality of life domains and three symptom scales [2]. They are valid and responsive tools (to therapeutic interventions) for measuring quality of life in both clinical and research studies [1,2]. However, the HRQOL scales are time-consuming and difficult to incorporate as a routine 'health status' tool for clinical practice. This is partly due to the comprehensive nature of the questionnaires. The average time to complete an HRQOL questionnaire for an adult CF patient is between 15 to 20 minutes [1,2]. The global scale (self-rated health status scale) has been used in other condition, for example, in patients with chronic obstructive pulmonary disease, to monitor patient's condition, and to predict future episodes of acute exacerbation and hospital readmission [5]. It takes a couple of minutes to complete. There is often very little time available in a busy outpatient clinic for adult CF patients to complete lengthy questionnaires and for the health care professionals' to supervise questionnaire completion.

We hypothesise that a single-item global quality of life scale may be potentially useful for routine clinical practice in outpatient settings for adult patients with cystic fibrosis. A reliable and valid single-item global quality of life scale is desirable because of its brevity. In addition self-completing by the patient avoids disrupting the flow of routine clinical care. It will also provide an alternative and/or complement the existing HRQOL scales for researchers and clinicians.

The rationale behind assessing the single item global scale in relation to the CF-QOL was to determine its psychometric property and the repeatability of the scale within two periods of time, in order to quantify the sensitivity and specificity of the scale. In addition, there are no 'gold standard' criteria to validate the single item global quality of life scale for adult patients with cystic fibrosis. Depressive and anxiety symptoms are common in adult CF patients [6]. We broadly hypothesised that a valid single-item global scale is most likely to relate to negative or positive psychological well-being (anxiety and depression) in adult CF patients. If our hypothesis is true then the lower score in the single item global quality of life scale (poorer global quality of life score) will be associated with impaired or poorer quality of life measured by CF-QOL [2] and with elevated levels of depressive and anxiety symptoms assessed by the Hospital Anxiety and Depression scale (HAD) scale [7].

We assessed the feasibility of administration the test-retest reliability and the validity of a single-item global quality of life scale. We also examined the relationship of the single item global quality of life scale with the disease-specific Cystic Fibrosis Quality of Life questionnaire (CF-QOL) [1], anxiety, depression and lung function with adult patients with cystic fibrosis.

## Methods

### Sample selection and procedures

Details of the methodology and other related data have been reported elsewhere [8]. Briefly, adult cystic fibrosis patients over 18 years of age were recruited from those attending routine outpatient clinics at a referral centre of the University teaching hospital, which covers the whole of the North-West of England. Participants were included if they had a confirmed diagnosis of CF and were able to read and speak English. Patients who were experiencing acute exacerbation or who had been admitted to hospital in the previous six-weeks were excluded. Lung function and other relevant data were extracted from the latest medical notes. The study protocol was approved by the local research ethics committee and all subjects gave informed consent to participate in the study.

The adult CF patients who provided informed consent were invited to complete the HAD scale [7], the CF-QOL [1] and single item global quality of life scale in the outpatient CF clinic. The researcher randomly administered the questionnaires, supervised completion and provided appropriate support when required.

The test-retest reliability of a measure is an estimate of its reproducibility over time when no change in condition has taken place. After ten days interval, all the outcome measures were sent by post with a pre-paid envelope to examine the test-retest reliability of the single-item global quality of life scale. All adult CF patients that completed the baseline outcome measures in outpatient clinic were sent the repeat questionnaires. An advisory letter was also sent to ensure that the health status of the adult CF patients was stable during this period. If they had an exacerbation, participants were advised not to complete the questionnaires.

### Measures

**Figure 1:**
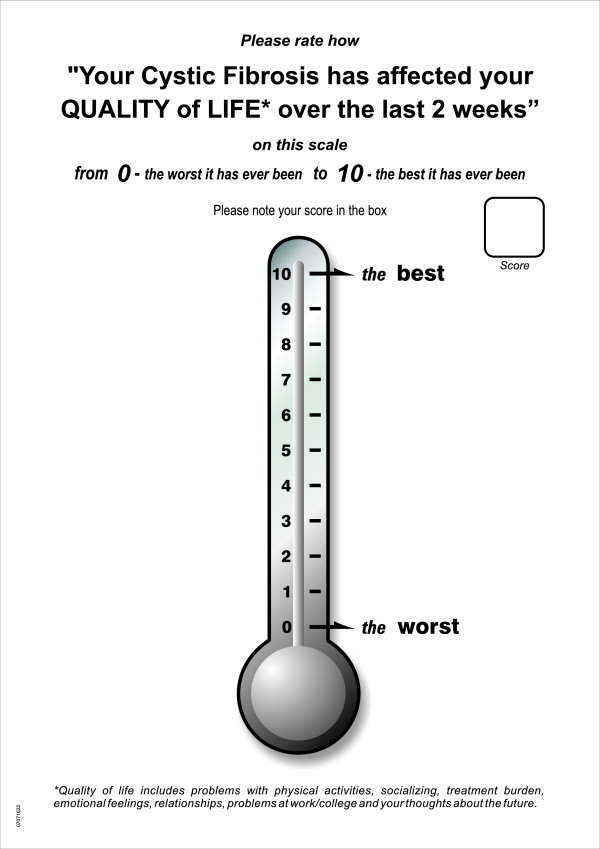
**The single item global quality of life scale for adult patients with cystic fibrosis**.

Adult CF patients rated their health status using a self-completed a single item global quality of life scale (with a barometer anchored, from 0 - 'the worst it has ever been' to 10 - 'the best it has ever been') over the last 2 weeks, see Figure 1. The subjects were shown in a vertical line scale with the numbers ranged from 0 to 10, with increments of 1. They were asked to rate the number that described the overall perception of their HRQOL. Lower scores on the single global quality of life scale indicate higher impact on adult CF patients' HRQOL.

Depression and anxiety in adult CF patients was measured by the HAD scale [7], which consists of 14 items (seven for anxiety and seven for depression). Each item is scored 0 to 3, with a maximum score of 21. It has established clinical cut-off scores: 0 to 7 normal symptom levels, 8 to 10 probable depressive or anxiety symptoms and ≥ 11 clinically-elevated for anxiety or depression [7,9]. The HAD scale has demonstrated excellent psychometric property of internal consistency coefficients (cronbach alpha) 0.78 for the anxiety sub-domain and 0.79 for the depression sub-domain [6,7]. The sensitivity and specificity of the HAD depressive or anxiety symptoms (at a cut off score ≥ 8, for each sub-domain) has been demonstrated above 0.80 in indentifying those with symptoms compared without symptoms [9].

The HRQOL was examined using a 52 item CF-QOL scale [1] across 9 domains of physical functioning, social functioning, treatment issues, chest symptoms, emotional functioning, concerns about the future, interpersonal relationships, body image and carer concerns. Response choices are ranged with a possible difficulty on a 6-point scale for performing an activity (e.g. I have had difficulty doing physical jobs. 1 = all of the time to 6 = never). Scores are standardised on a 0 to 100-point for each domain, with higher scores corresponding to better quality of life. The scale also gives an average aggregate score for all the domains. The internal consistency of the domains of the CF-QOL has been demonstrated with Cronbach alpha coefficients ranging between 0.72 - 0.92 [1]. The test-retest reliability of the CF-QOL for all domains is between (r = 0.74 to 0.96) [1]. The physical functioning domain of the CF-QOL demonstrated strong predictor of survival in patients with cystic fibrosis [10].

### Data analysis

Descriptive statistics such as percentages, means, medians, standard deviations and range were used where appropriate. The repeatability of the single-item quality of life scale and CF-QOL were assessed by the intra-class correlation coefficient [11] and limits of agreement [12]. We employed the conventional interpretation of the inter-class correlation coefficient: i.e. those values of 0.40 - 0.75 are fair to good and values over 0.75 are excellent [13]. The concurrent validity of the single item global scale was examined in relation to anxiety, depression, lung function and the domains of CF-QOL using Pearson Correlation coefficient tests. Differences between the groups of adult CF patients who did and did not complete the second assessment were examined using the Student t-test, the Mann-Whitney U-test and the chi-square test as appropriate. We examined the area under the receiver operator characteristic curve to assess the discriminative ability of a single-item global quality of life scale in relation to the CF-QOL scale and calculated sensitivity and specificity values. Significance was set at the conventional 5% level.

## Results

### Patient population

One-hundred twenty one adult cystic fibrosis patients (66 men and 55 women) completed the baseline measurements. Their mean (SD) age was 29.6 (8.9) years and mean FEV_1_(SD) was 2.20 (0.94) litres. Of these, 33 cystic fibrosis patients (17 women) completed the post-two-weeks measurement. The mean (SD) age in the group retested was 32.0 (10.2) years compared to 28.6 (8.2) years in those adult CF patients who were not retested.

Figure 1 shows the single item global quality of life scale for adult patients with cystic fibrosis.

**Figure 2:**
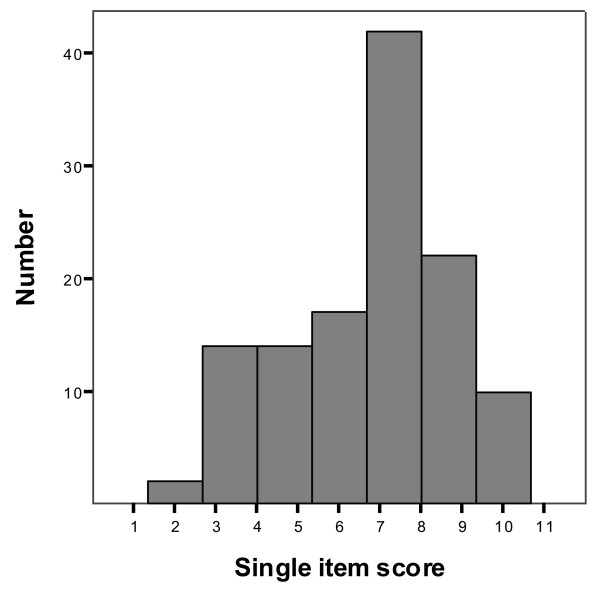
**The distribution of the single-item score for 121 adult cystic fibrosis patients**.

Figure 2 shows the distribution of the single-item score at baseline.

### Representativeness of the sample with repeat measures

There are no statistically significant differences between age, gender, single item score, health related quality of life, anxiety and depression those who completed (n = 33) the single item and those who did not respond (n = 88) the repeatability questionnaires see Table 1.

**Table 1:** Establishing the representativeness of the sample of 33 with repeat questionnaire information

	Repeat questionnaire (N = 33)	No repeat questionnaire (n = 88)	p-value
Gender % male	48%	57%	0.54
Single item; mean(sd)	6.9 (1.8)	6.9 (2.0)	0.83
CF-QOL; mean(sd)	71.4 (14.3)	73.0 (16.4)	0.63
Age; mean (sd)	32.0 (10.1)	28.6 (8.2)	0.07
HAD anxiety; median (range)	6 (0 to 14)	5 (0 to 16)	0.24
HAD depression; median (range)	2 (0 to 11)	2 (0 to 13)	0.88

HAD = Hospital anxiety and depression scale

CF-QOL = Cystic fibrosis quality of life scale

### Repeatability for single item global scale (baseline versus 2 weeks)

The Intra-class correlation coefficient for the single item global scale was 0.78; 95% CI (0.59 to 0.88). The mean difference between the two readings (2^nd^-1^st ^reading): -0.1; 95% limits of agreement = -2.5 to 2.3. Hence the discrepancy between two readings could be as large as 36% (= 2.5/6.9). Of the 33 subjects, 18 (55%) had identical readings, 28 (85%) had readings with a discrepancy of 1 or less. The greatest discrepancy (for one subject) was 4.

### Repeatability for CF-QOL (baseline versus 2 weeks)

The intra-class correlation coefficient for the CF-QOL was = 0.83; 95% CI (0.68 to 0.91). The mean difference between the two readings was (2^nd^-1^st ^reading): 1.4; 95% limits of agreement = -15.1 to 17.9. The discrepancy between the two readings could be as large as 25% (= 17.9/72). Of the 33 subjects, 21 (64%) had readings with a discrepancy of 5 or less, 27 (82%) had readings with a discrepancy of 10 or less. The greatest discrepancy (for 1 subject) was 25.

### Concurrent validity of the single item global quality of life scale

Table 2 shows the correlation of the single item global scale with CF-QOL domains, anxiety and depression and with forced expiratory volume in one second (FEV_1_). Most of the CF-QOL variables were moderately correlated (r = 0.38 - 0.61, p < 0.001) with the single item global scale. The single-item global scale was weakly correlated with FEV_1 _and body image (r = 0.21 and r = 0.25, p = 0.01, respectively). Higher scores with the single-item global scale was correlated negatively with anxiety (r = -0.50, p < 0.001), depression (r = -0.38, p < 0.001) and frequency of hospital readmission in the previous year (r = -0.39, p < 0.001).

**Table 2:** Correlation of single item quality of life with CF-QOL, anxiety and depression, FEV_1_

CF-QOL scale	Single item quality of life scale	p-values
Physical functioning	r = 0.46	< 0.001
Social functioning	r = 0.51	< 0.001
Treatment issues	r = 0.41	< 0.001
Chest symptoms	r = 0.50	< 0.001
Emotional functioning	r = 0.61	< 0.001
Concerns about the future	r = 0.43	< 0.001
Interpersonal relationships	r = 0.56	< 0.001
Body image	r = 0.25	= 0.01
Carer concerns	r = 0.53	< 0.001
Total QOL score	r = 0.64	< 0.001
Anxiety	r = -0.50	< 0.001
Depression	r = -0.38	< 0.001
FEV_1_	r = 0.21	= 0.02

CF-QOL = Cystic fibrosis Quality of Life scale

FEV_1 _= Forced expiratory volume in one second

### Sensitivity and specificity of a single item global quality of life scale

**Figure 3:**
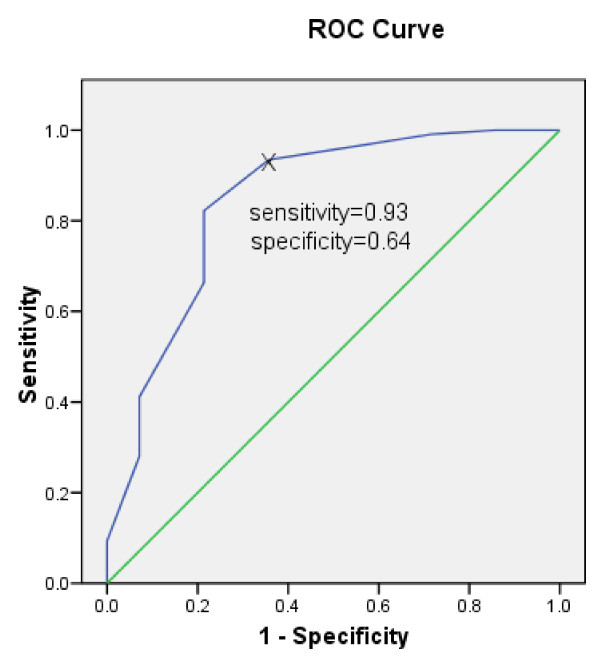
**The sensitivity and specificity of the single-item global quality of life scale compared to a 50% threshold for the cystic fibrosis quality of life scale**. The single item global quality of life score predicts of the adult cystic fibrosis patients with the cystic fibrosis quality of life score > 50. The receiver operating characteristic area was 84.

Figure 3 shows the predictive ability of a single-item scale related to a health related quality of life score > 50. One-hundred and seven (88%) subjects had a CF-QOL score > 50. The optimal threshold for a single item global scale for score 5 or more showed sensitivity = 93% (100/107), 95% confidence interval (87% to 97%) and specificity 64% (9/14), 95% confidence interval (39% to 84%). The likelihood ratio for a positive test (LR+) was 2.6; 95% confidence interval (1.3 to 5.3). Increasing the threshold of a single item global scale by 1 point to 6 or more reduced the sensitivity to 82% (88/107) and increased the specificity to 78% (11/14). The area under the ROC curve was 0.84.

**Figure 4:**
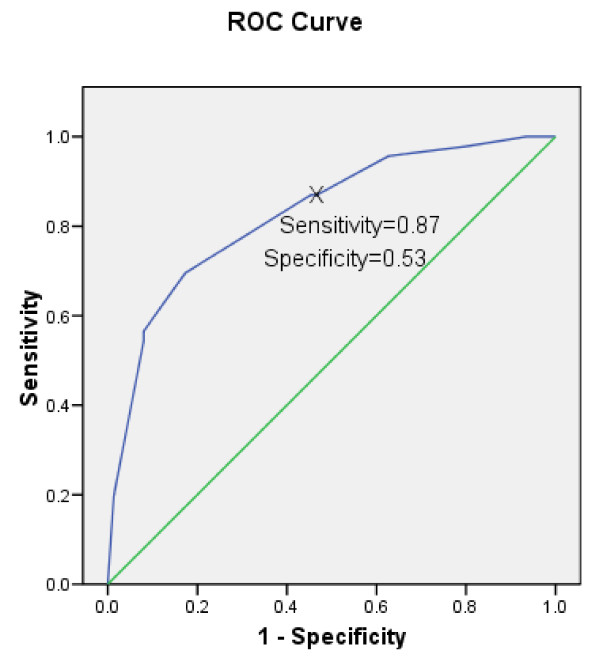
**The sensitivity and specificity of the single-item global quality of life scale compared to a 80% threshold for the cystic fibrosis quality of life scale**. The single item global quality of life score predicts of the adult patients with the cystic fibrosis quality of life score > 80. The receiver operating characteristic area was 83.

Figure 4 shows the predictive ability of a single item scale related to a health related quality of life score > 80. Forty-three (33%) of subjects had a CF-QOL score > 80. The optimal threshold for a single item for score 7 or more showed sensitivity = 87% (40/46), 95% confidence interval (74% to 94%) and specificity = 53% (40/75), 95% confidence interval (42% to 64%). The likelihood ratio for a positive test (LR+) was 1.9; 95% confidence interval (1.4 to 2.4). Increasing the threshold of a single item global scale by 1 point to 8 or more, the sensitivity value reduced to 70% (32/46), and the specificity value increased to 83% (62/75). The area under the ROC curve was 0.83.

## Discussion

This is the first study to show that the single-item global quality of life scale demonstrate evidence of the reliability and validity for measuring the health status of adult patients with cystic fibrosis. The single item global quality of life scale has the ability to distinguish between adult CF patients with lower CF-QOL scores compared from those with higher scores. The main potential advantage of the single-item global quality of life scale compared to other CF-QOL measures is its brevity and applicability for use in routine clinical practice.

The interclass correlation coefficients for both the single-item global quality and CF-QOL (0.78 and 0.83, respectively) were very high (values greater than 0.75 are excellent) as determined previously [13] showing high reliability of the scales. The 95% confidence intervals for the intra-class correlations are reasonably narrow. In addition, there was little evidence of a biased group of subjects with information on two sets of questionnaires. Those subjects with repeat questionnaires had similar QOL scores and HAD scores to those without repeat questionnaires. They were slightly older (but only by an average age of 3 years) and had a slightly lower percentage of males.

The repeatability scores between measures over 2 weeks revealed that there was less variability of total CF-QOL scores, 25% compared with 36% for the single item global scale. This signifies that the disease-specific CF-QOL scale with 9 domains was more stable compared with the single-item global scale, which was similar finding that had been reported in other chronic respiratory disease [14].

The sensitivity and specificity scores were relatively high for both definitions of poor/good health using the CF-QOL scale, showing that the single item quality of life score was a reasonably accurate predictor of health related quality of life.

There is no 'gold standard' outcome measure for assessment of quality of life in adult cystic fibrosis patients. However, there are valid disease-specific quality of life scales for adult patients with cystic fibrosis [1,2]. The concurrent validity (a test against well-established measures) of the single item scale was examined in relation to quality of life using the CF-QOL scale [1], psychological well-being (anxiety and depression) [8] and lung function tests. Findings demonstrated that the single item global quality of life scale was moderately better correlated with the some of the domains that are related to psychosocial, physical functioning and chest symptoms, while in others it was weakly correlated with the body image and lung function as reported in Table 2. The potential reasons for these variations are unclear. For instance, the weaker correlation between the single item quality of life scale measure and body image may suggest that adult patients with CF may have adopted a level of negative image (stigma) of the disease in manner that is different from an adaptation to physical functioning. This is in contrast to previous findings study [3] that body image explained 30% of the variance in the CF-QOL score. The association of frequent episodes of hospital admission with impaired quality of life partly may be explained that some of the adult CF patients may experience frustration with the disease and inability to cope at home in turn may lead them to social isolation and poorer physical health status.

Our data is also in agreement with previous studies in patients with chronic obstructive pulmonary disease [5,14], the low correlation between FEV_1 _and the single item quality of life scale value is partly may be explained by the limited ability of FEV_1 _as a measure of disease severity in CF [15]. However, the single item global scale was moderately correlated with the anxiety and depression scores. It may signify the importance of psychological well-being for the individual patient and provide better estimates of quality of life adult patients with cystic fibrosis. Further study is required to demonstrate the responsiveness of the scale to an intervention in a clinical setting.

Several limitations of the present study are noteworthy. Firstly, the study was conducted in a single referral centre in outpatient clinic in which the majority of the patients were Caucasian. Thus, the generisability of the findings to different demographics requires further investigation. Due to the cross-sectional nature of this study, we cannot hypothesize whether the single-item global scale is responsive to change over time. Further validation is required for the use of the scale for epidemiological or longitudinal surveys. Secondly, the postal questionnaire has a disadvantage in terms of uncertainty who completed the questionnaire (whether it was completed by patients or carers). Less than one-third of the CF patients responded to our postal questionnaire a poor response rate introducing the potential for response bias i.e. motivated individuals may be more likely to participate in this study. However, there was no significant difference between those who responded and those who did not respond in terms of socio-demographic characteristic data (Table 1). Furthermore, we performed robust statistical tests for the scale validation using the Interclass-correlation coefficient and the receiver operating characteristic analyses. Finally, the relative small sample size requires replication in a larger study. In addition, the single-item global quality of life scale provides clinicians with limited information about the patient health status but acts as a screening tool. Therefore, detailed investigation is most likely to be desirable to those patients who responded low scores with the single-item quality of life scale: 'this must be balanced against the practicality of ascertaining such information. Brevity may come at a cost of detail.' [16].

### Clinical implication

We believe that the single item global scale is a promising tool that can be incorporated in clinical environments to assess adult cystic fibrosis patients' quality of life. It is simple to administer in routine clinical practice in an outpatient setting as it would not be burdensome (time-consuming) for the adult cystic fibrosis patients to complete. In addition, it will help to identify early those adult cystic fibrosis patients with a 'health status worsening' in order to provide and target appropriate intervention.

## Conclusions

The single-item quality of scale is acceptable, valid and repeatable for adult patients with cystic fibrosis. Further studies are needed to fully validate the single-item quality of life scale in a larger sample size and assess its responsiveness to interventions.

## Competing interests

The authors declare that they have no competing interests.

## Authors' contributions

AMY has taken main responsibility for the study's data collection, analyses, interpretation of the results, and in writing the first draft and subsequent revision of the manuscript. MD participated in the study design, data collection, conduct of the study and the editing of the article. JM contributed to the statistical analyses and editing of the manuscripts. KW participated in the study design, preparation and the editing of the article. All authors have read and approved the final manuscript.
